# A Guided Tour of the Structural Biology of Gaucher Disease: Acid-**β**-Glucosidase and Saposin C

**DOI:** 10.4061/2011/973231

**Published:** 2011-11-22

**Authors:** Raquel L. Lieberman

**Affiliations:** School of Chemistry & Biochemistry, Institute for Bioscience and Bioengineering, Georgia Institute of Technology, 901 Atlantic Drive NW Atlanta, GA 30332-0400, USA

## Abstract

Mutations in both acid-**β**-glucosidase (GCase) and saposin C lead to Gaucher disease, the most common lysosomal storage disorder. The past several years have seen an explosion of structural and biochemical information for these proteins, which have provided new insight into the biology and pathogenesis of Gaucher disease, as well as opportunities for new therapeutic directions. Nearly 20 crystal structures of GCase are now available, from different heterologous sources, complexed with different ligands in the active site, in different glycosylation states, as well as one that harbors a prevalent disease-causing mutation, N370S. For saposin C, two NMR and 3 crystal structures have been solved, each with its unique snapshot. This review focuses on the details of these structures to highlight salient common and disparate features that contribute to our current state of knowledge of this complex orphan disease.

## 1. Introduction

Gaucher disease (GD) is a human catabolic disorder mainly due to mutations in the gene encoding for the lysosomal enzyme acid-*β*-glucosidase (GCase) [1]. As a consequence of an amino acid substitution in the resultant protein, its major substrate, N-acyl-sphingosyl-1-O-*β*-D-glucoside (GlcCer), accumulates, is engulfed in macrophages, and clinically results in enlarged organs, splenomegaly, hepatomegaly, and, in severe Gaucher cases, disorders of the central nervous system and brain. GD occurs in 1 : 10000 births in the general population but is much more prevalent in the Ashkenazi Jewish population, where its incidence is estimated as high as 1 : 200 [1]. Therapies only exist for non-neuronopathic Gaucher disease [2], which include enzyme replacement therapy using recombinant enzyme [3], and substrate reduction therapy using a small molecule inhibitor of a GlcCer biosynthetic enzyme [4]. 

Mature GCase is a glycoprotein consisting of 497 amino acids derived from a precursor that is proteolyzed prior to lysosomal trafficking [5, 6]. The wild type enzyme is trafficked via a pathway independent of the more typical mannose receptor pathway [7] in a recently discovered association with lysosomal integral membrane protein II (LIMP-II) [8]. However, since patients with mutations in LIMP-II do not exactly replicate Gaucher symptoms [9], other lysosomal trafficking pathways for GCase may also exist. By comparison, recombinant, therapeutic, GCase is engineered for lysosomal targeting via the mannose receptor pathway and is decorated with mannose-6-phosphate (M6P) [10].

Almost 300 different point mutations GCase are known to cause GD, by far the most prevalent of which are missense mutations [11]. When an amino substitution is introduced, mutant GCase is retained in the ER where it is targeted to degradation, leading to a reduction in enzyme levels [12–15]. However, when expressed in the laboratory, many mutant GCase variants, such as N370S predominant among Ashkenazi Jews, produce stable enzymes with residual, albeit impaired, activity [16]. These findings have fueled new therapeutic efforts to decrease degradation and rescue mutant GCase lysosomal trafficking with small molecules, which may cross the blood brain barrier and be therapeutic for neuronopathic GD [17–20].

GCase falls into the large family of glycoside hydrolases, well-studied enzymes found throughout biology that use catalytic aspartate or glutamates for general acid/base hydrolysis [21]. Human lysosomal GCase belongs to the GH30 family (http://www.cazy.org/Glycoside-Hydrolases.html) [22], enzymes in which use a chemical mechanism to retain the strereochemistry of the substrate. For GCase, the catalytic nucleophile is Glu 340, identified from a mass spectrometric adduct using a covalent inhibitor [23], and the general acid/base residue is Glu 235, identified unambiguously from the GCase structure [24]. The mechanism for retaining *β*-glucosidases [25, 26] uses double-displacement acid/base chemistry involving nucleophilic attack of a deprotonated glutamate to form a glycosyl-enzyme intermediate, followed by hydrolysis of the adduct. This reaction is proposed to proceed through two oxocarbenium ion-like planar transition states [21], and the pKa of each carboxylate tailors the side chain for its particular function [27, 28]. In vitro, GCase has been assayed using radiolabeled GlcCer and thin liquid chromatographic separation [28] or model fluorescent substrates [29]. The wild-type GCase sources discussed in this review exhibit a *K*_*m*_ for various substates in the low to mid *μ*M range, and *V*_max_ of ~0.5 *μ*M/min [29, 30]. Among disease-causing mutants tested [16, 31], the turnover number, *k*_cat_, is lower than wild-type GCase, and, consistent with this observation, the N370S-mutant GCase specifically exhibits a reduced *V*_max_ and increased *K*_*m*_ [32].

In the lysosome, the wild-type GCase is membrane-associated and requires the activator protein saposin C (SapC) for catalysis [39]; mutations in SapC also lead to GD [40, 41]. Originally isolated from the spleen of Gaucher patient [39], SapC derives from a prosaposin cursor [42] and increases substrate hydrolytic rates of GCase in vitro [40, 43, 44]. This process is both reversible and pH-controlled [39, 45]. SapC remodels the lipid membrane [46, 47], presumably to assist GCase in accessing the short headgroup of GlcCer, likely via a multistep mechanism [48, 49]. In particular, SapC is believed to modulate the lysosomal membrane structure in a detergent-like solubilizing manner [50]. Recent atomic force microscopy and other spectroscopic studies reveal that GCase associates with SapC at the membrane surface [46], although its explicit binding modes are not well-understood.

Many excellent reviews exist on GCase and SapC biochemistry, as well as clinical aspects of GD including its current and future treatment. Several are listed here and throughout this document for further reading [1, 2, 51–59]. This review focuses on a comparison of the available structures of GCase and SapC and their contributions to our current state of knowledge of the biology and pathogenesis of this heterogeneous orphan disease.

## 2. Structure of GCase

### 2.1. Protein Sources

To date, nearly 20 crystal structures of GCase have been solved under different conditions, including 12 more since the first review article on GCase structures was published in 2008 [53]. The source of enzyme is the same as that used in, or in development for, patient treatment: Cerezyme (purified from CHO cells), Taliglucerase-alfa (purified from carrot cells, prGCase), or Velaglucerase-alfa (purified from human cell line). The first two sources have an inocuous single mutation introduced near the C-terminus, R495H, not present in Velaglucerase-alfa [30]. The first structure of mutant GCase, N370S, expressed in baculovirus, became available in late 2010 [32]. A list of GCase structures available in the protein databank (PDB) (http://www.rcsb.org/) at the time of the writing of this review is presented in Table 1.

### 2.2. Crystallization Conditions

Three main crystallization conditions have been reported (Table 1). The first two, utilized to obtain the majority of structures, including all those for Cerezyme and for Velaglucerase-alfa, employs vapor diffusion and similar high-salt cocktails. One condition uses low pH and molar concentration of ammonium sulfate [24] whereas the second uses near molar concentrations of phosphate and a variety of pH buffers [35]. Both of these conditions lead to crystals with a lattice of approximate dimensions 109 Å × 285 Å × 91 Å that belong to either the orthorhombic (all angles 90°) space group C222_1_ with two independent copies of GCase in the asymmetric unit or the monoclinic (*β* angle = ~109°) P2_1_ with four such GCase molecules in the asymmetric unit. The lattice selection depends on how the enzyme is packed in the crystal and determines which molecules are considered equivalent by symmetry. Thus, in C222_1_, the higher symmetry space group, there is additional averaging over molecules in the unit cell compared to the case of P2_1_, leading to 2 or 4 independent views of GCase for each reported structure, respectively. The first GCase structure was solved by obtaining experimental phases from a bound mercury ion [24], whereas subsequent structures have been solved either by molecular replacement of this initial structure [29, 30, 32, 34, 35] or by rigid body refinement in the case of isomorphous crystals [33, 36–38]. The third crystallization condition uses the microbatch method under oil and contains polyethylene glycol 3350 as the main precipitant [36]. Thus far, only prGCase appears to crystallize using this cocktail, which has been particularly successful in capturing structures with hydrophobic active-site-directed inhibitors (see what follows) [36, 37]. In this case, the lattice belongs to the space group P2_1_ with two independent views of GCase in the asymmetric unit and lattice parameters of approximately 68 Å, 97 Å, 83 Å, *β* = 104°.

### 2.3. Overall Structure

**Figure 1:**
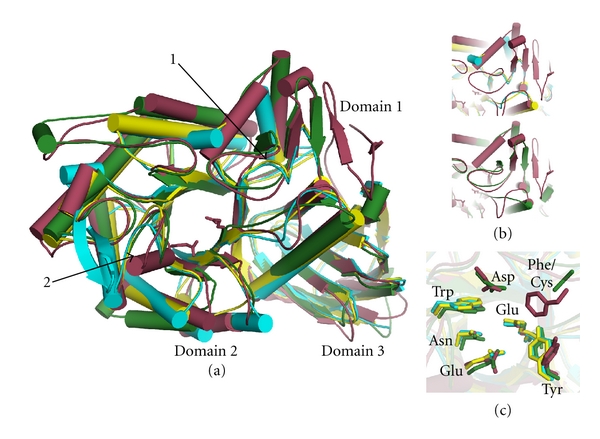
Superposition of GH30 family member structures. Cartoon representation: apo GCase (PDB code 3GXD), raspberry red; xylanases, yellow (PDB code 1NOF) and blue (PDB code 3LK0); SrfJ, green (pdb code 2WNW). (a) Overall structures with domains labeled. Arrows 1, 2: deviations in Domains 1 and 2, respectively (see text). (b) Top: comparison of GCase and xylanases (blue, yellow) in Domain 1 region; bottom: comparison of GCase and SrfJ (green) in Domain 1 region. (c) Active site region (Domain 2) with select amino acid side chains depicted in ball-and-stick.

**Figure 2:**
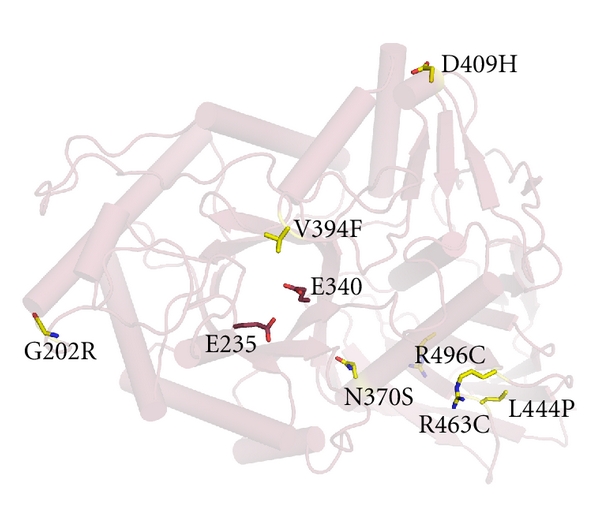
Location of just six of over 200 missense mutations known to cause GD mapped onto the GCase structure. Yellow: site of amino acid substitution; raspberry red: catalytic residues Glu 235 and Glu 340.

Regardless of crystallization condition, molecules in asymmetric units from all GCase sources exhibit root mean squared differences (rmsd) of ~0.6 Å, indicating that the views of GCase are nearly identical. GCase comprises three discontinuous domains (Figure 1(a)): an antiparallel *β*-sheet (Domain 1), a triose phosphate isomerase (TIM) barrel harboring the active site (Domain 2), and an 8-stranded *β*-barrel (Domain 3). Domains 2 and 3 are seen in similar relative orientations in other hydrolases, such as in *α*-galactosidase A, mutations in which cause another lysosomal storage disorder, Fabry disease, even in the absence sequence similarity [38]. Importantly, as in *α*-galactosidase A [60], mutations are found throughout the three-dimensional structure of GCase and are not localized to a particular patch on the enzyme [24] (Figure 2 and see the following discussion). 

Just four different enzyme structures are available for the GH30 glycosidase family. Domains 2 and 3 are common to these orthologs (Figure 1(a)), but the structures diverge in the region of Domain 1 (Figure 1(a), arrow 1) and its immediate environs (Figure 1(b)). For both GH30 xylanases solved to date (pdb code 3KL0s [61], 1NOF [62], formerly members of GH5 family [61]), Domain 1 is absent, and a nearby loop that covers the GCase active site (see what follows) is also missing (Figure 1(b), top panel). A more similar structural homolog (PDB code 2WNW), *S. typhimurium* SrfJ involved in bacterial pathogenicity [63], has a truncated Domain 1. A *β*-hairpin is followed by the aforementioned GCase loop (Figure 1(b), lower panel), albeit with low sequence conservation (see Figure 1(c), Phe/Cys substitution). The precise substrate of SrfJ remains to be elucidated, but it seems likely that SrfJ may recognize alkyl chain containing glycosides, similar to GCase [63]. One region unique to GCase is near the presumed general acid-base residue Glu 235 (see the following), which takes on a helical conformation. All three other GH30 structures do not have defined secondary structure features in this region (Figure 1(a), arrow 2). Although implications for catalysis are not known, the proximity to the active site, which is otherwise very similar and highly conserved (Figure 1(c)), suggests that this region may assist in tuning chemistry in the active site.

### 2.4. Active Site

**Figure 3:**
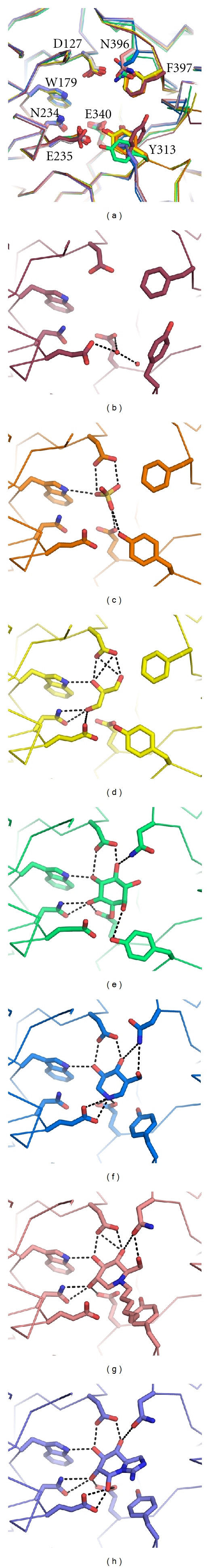
Active sites from different GCase structures. (a) Superposition of structures presented in (b)–(h) with residues labeled and any ligands omitted. (b) Apo GCase, (c) sulfate-bound GCase (PDB code 1OGS), (d) glycerol-bound GCase (PDB code 1NT1), (e) CBE-bound GCase (PDB code 1Y7V), (f) IFG-bound GCase (PDB code 1NT1), (g) NB-DNJ (PDB code 2V3D), and (h) N-octyl(cyclic guanidine)-nojirimycin (PDB code 2WCG). Dashed lines indicate hydrogen bonding interactions (2.5–3.5 Å distance from N, O atoms).

At minimum, the active site consists of the residues known to be involved in catalysis, namely, Glu 340, the nucleophile, Glu 235, the presumptive general acid-base residue located 5 Å away from Glu 340, plus residues involved in stabilizing GlcCer in the active site. This region encompasses both a well-defined binding site for the glucose moiety within Domain 2 (Figure 1(a)) whereas the ceramide region is less well understood (see the following discussion below). Residues that line the glucose-binding region but are not directly involved in catalysis include Arg 120, Asp 127, Phe 128, Trp 179, Asn 234, Tyr 244, Phe 246, Tyr 313, Cys 342, Ser 345, Trp 381, Asn 396, Phe 397, and Val 398 (Figure 3(a); some residues are omitted from the image for clarity). The aromatic side chains are thought to be involved in substrate recognition [64] and several polar residues form hydrogen bonding interactions with substrate. Many of the residues are located on the interior of GCase within well-defined secondary structural elements and remain essentially static regardless of what may be bound in the active site. Other residues, including Tyr 313, Asp 315, Asn 396, and Phe 397, among others, are sensitive to ligand binding, as discussed below. In the apo GCase active site, these residues have high thermal B-factors, indicating that crystallographically related residues in this region of the protein sample a number of different conformations, and Figure 3(b) is just an average of several accessible to enzyme [35, 38].

As a result of a component in the crystallization cocktail, two ligands have serendipitously appeared bound in the active site. A sulfate anion was modeled in the active site of the first GCase structure [24]. A rationale for this assignment is the high concentrations of ammonium sulfate in the crystallization condition. Assuming that the sulfate anion is partially protonated, which is likely given the pH of the crystallization condition, hydrogen bonding interactions with catalytic residue of GCase are present (Figure 3(c)). In a later structure at slightly higher resolution but using the same crystallization conditions, an uncharged, polar glycerol molecule is modeled in the active site [35] (Figure 3(d)). Glycerol is present at 20% in the mother liquor used to protect the crystal upon cryo-cooling before data collection and a characteristic “w” shape of glycerol was clearly apparent in difference density maps [35]. Like what is expected for the hydroxyl substituents of glucose, glycerol is stabilized by hydrogen bonding interactions with polar residues in the active site (Figure 3(d)). It is possible that differences in cryo-cooling procedures led to different molecules bound in the GCase active site. A “true” apo GCase was achieved by using Li_2_SO_4_ for cryprotection instead of glycerol [38]; in this structure only waters appear in the active site, and the GCase scaffold remained essentially unchanged (Figure 3(b)).

GCase crystal structures have been solved with numerous intentional ligands as well, to investigate conformational changes in the active site that may arise upon their binding and help exploit this knowledge for small molecule drug development [65, 66]. In the first such investigation, the known suicide inhibitor, 1,2-anhydro-*myo*-inositol (CBE), was added to preformed GCase crystals [33] (Figure 3(e)). Though the overall enzyme structure is nearly identical to previous structures, several features are seen in the active site. Most importantly, the observed adduct firmly established that enzyme inactivation by CBE is a result of its binding to the active site, and in particular, its covalent attachment to nucleophile Glu 340 and not to any other residues [33], confirming previous mass spectrometry data [23]. The structure is also consistent with the proposed enzymatic mechanism, which involves protonating the epoxide oxygen by Glu 235 followed by nucleophilic attack of the *myo*-inositol ring by Glu 340, forming the nucleophile-*myo*-inositol ester bond [67]. The once-epoxide oxygen is pointed toward Glu 235 in the product* myo*-inositol but might also be stabilized by hydrogen bonding interactions with Asn 234 (not shown). Unexpectedly, the product is in a boat conformation where a chair conformation was expected [33]. Last, it is in this structure that Asn 396 was first observed in the active site of GCase, replacing the position of Phe 397, where it assists in holding *myo*-inositol in place.

Subsequent work has revealed GCase bound to reversible iminosugar inhibitors including isofagomine (IFG) [35], N-butyl and N-nonyl deoxynojirimycin (NB- and NN-DNJ, resp.) [36], and the bicyclic fused ring 6-amino-6-deoxy-5,6-di-*N*-(*N *′-octyliminomethylidene) nojirimycin [37] (Figures 3(f)–3(h)). Whereas the structure of GCase with IFG was solved by soaking the compound into a crystal of deglycosylated Cerezyme, the latter structures were obtained by cocrystallization with prGCase under oil. No global changes are observed in the GCase structure upon compound binding, but several changes are observed in these structures that provide insight into a likely mode for GlcCer binding. First, in all four structures, three of which are presented in Figure 3, the compounds are held in the active site by extensive hydrogen bonding interactions with the hydroxyl and hydroxymethyl substituents (Figures 3(f)–3(h)). Compared to the IFG and DNJs (Figures 3(f) and 3(g)), the bicyclic analog (Figure 3(h)) lacks the hydroxymethyl arm, and instead, a fourth hydroxyl group is within hydrogen bonding distance of Glu 235. In all cases, Asn 396, but not Phe 397, is present in the active site and participates in stabilizing the inhibitor. Second, compared to the sulfate or glycerol-bound structures (Figures 3(c) and 3(d)) the position of Tyr 313 has moved and is now in hydrogen bonding distance of Glu 340 instead of Glu 235. Third, the placement of the endocyclic nitrogen in each of these compounds is informative. The secondary amine present in the piperidine ring of IFG appears to mimic the position of the anomeric carbon of GlcCer and positions Glu 340 and Glu 235 for hydrolysis. Thus, IFG is a candidate transition state inhibitor [68] or product mimic [35]. Notably, due to its high pKa of 8.4 [69], IFG is likely protonated. By contrast, the tertiary amines found in the DNJ analogs are shifted with respect to the amine of IFG. The position of amines in DNJ and bicyclic analogs mimic the endocyclic oxygen of the glucose headgroup of GlcCer, and they do not make any contacts with GCase. The pKa of this nitrogen is ~7 [36] and thus may also be protonated at low pH of the lysosome and crystallization condition. The configuration of the bound DNJs indicates that they are not transition state mimics for GCase. Lastly, positions of the hydrophobic tails are also of interest, as they mimic the ceramide portion of GlcCer. The alkyl tails of NB- and NN-DNJ appear to be stabilized by interactions with Tyr 313 and another hydrophobic residue outside the immediate active site, Leu 314 (not shown) [36]; unfortunately, no electron density was visible for the alkyl chain of the fused bicyclic analog for comparison [37].

On the basis of the GCase structures with bound inhibitors, some details of GCase catalysis can be confirmed. First, Glu 235 and Glu 340 are separated by 5 Å, as expected of a retaining glycosidase [21]. Second, whereas direct nucleophilic attack of Glu 340 on the anomeric carbon of GlcCer has been suggested [21], in the IFG-bound GCase structure, the piperidine nitrogen is 2.7 Å away from Glu 340 and Glu 235, a length more consistent with hydrogen bonding. Direct attack of a Glu 340 might be prevented by the presence of the apical hydrogen on the anomeric carbon in this position of GlcCer [36], but it is certainly possible that IFG is not a suitable analog to investigate such mechanistic details. By contrast, nucleophilic attack on CBE can be envisioned more readily because the hydrogen atom is not apical, thus reducing steric hindrance [33]. Third, a planar intermediate is also anticipated [25]. As observed with the bicyclic inhibitor [37], this can be accommodated readily in the active site. In spite of these views of bound inhibitors, several questions remain open for investigation, including the protonation state of Glu 235 as well as the potential role of water in catalysis [36]. 

### 2.5. Loops in the Vicinity of the Active Site

**Figure 4:**
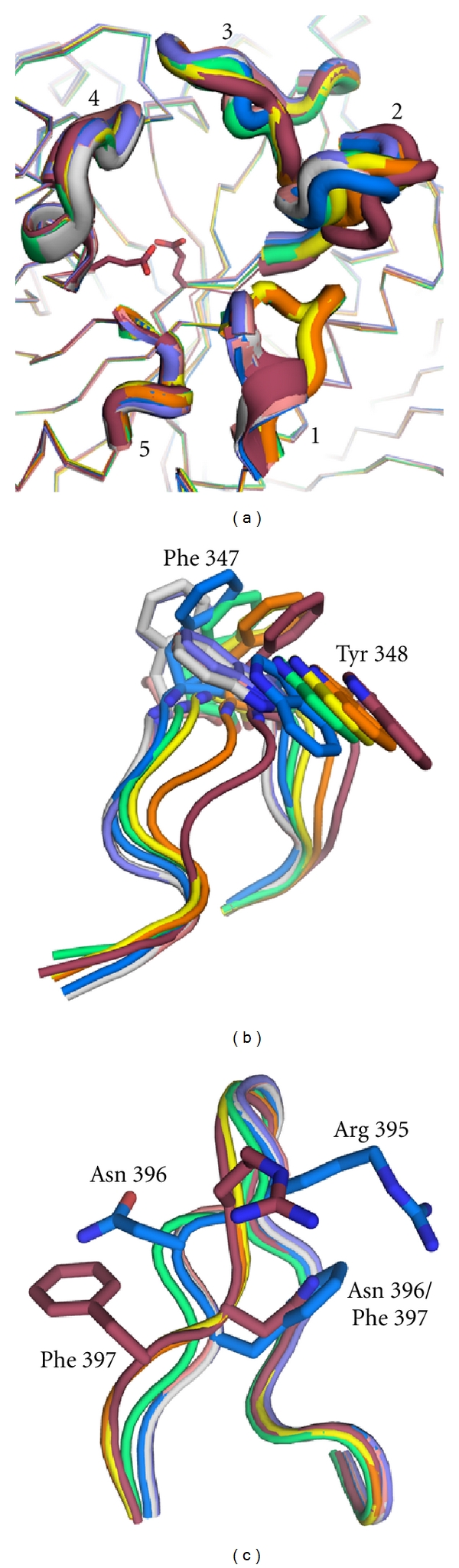
Superposition of GCase loops 1–5 in vicinity of active site. (a) Overlay of all loops, (b) detailed comparison of loop 2, and (c) detailed comparison of loop 3. Colors are the same as in Figure 3 with the addition of grey for NN-DNJ (PDB code 2V3E).

Whereas the site of catalysis is well-defined in GCase, the hydrophobic binding sites for ceramide are less clear. Indeed, the need for such a binding site is not obviously necessary, given the proximity of GCase to the lysosomal lipid membrane, but specific subsites were predicted from studies of human spleen-derived GCase [28]. Initially, based on the first crystal structure, which lacked a hydrophobic surface for such binding, it was proposed that the glucose moiety is bound in the active site, with ceramide protruding out of the protein and into the presumed lipid bilayer [24]. Nevertheless, the presence of five loops (Figure 4), Loop 1 (residues 311–319), Loop 2 (residues 345–349) and Loop 3 (residues 394–399), but also Loop 4 (237–248) and Loop 5 (283–288) capping the active site, suggested that rearrangements might be possible and, if so, could reveal a new binding site. The extent of the mobility of these loops appeared minor at first, however. GCase structures solved to date overlay particularly well in loop 1 and loop 5 (Figure 4(a)), and crystal contacts in loop2 (Figure 4(b), Phe 347- Trp 348) and loop 2 (Figure 4(c)) may preclude the observation of fluctuations that take place in solution.

**Figure 5:**
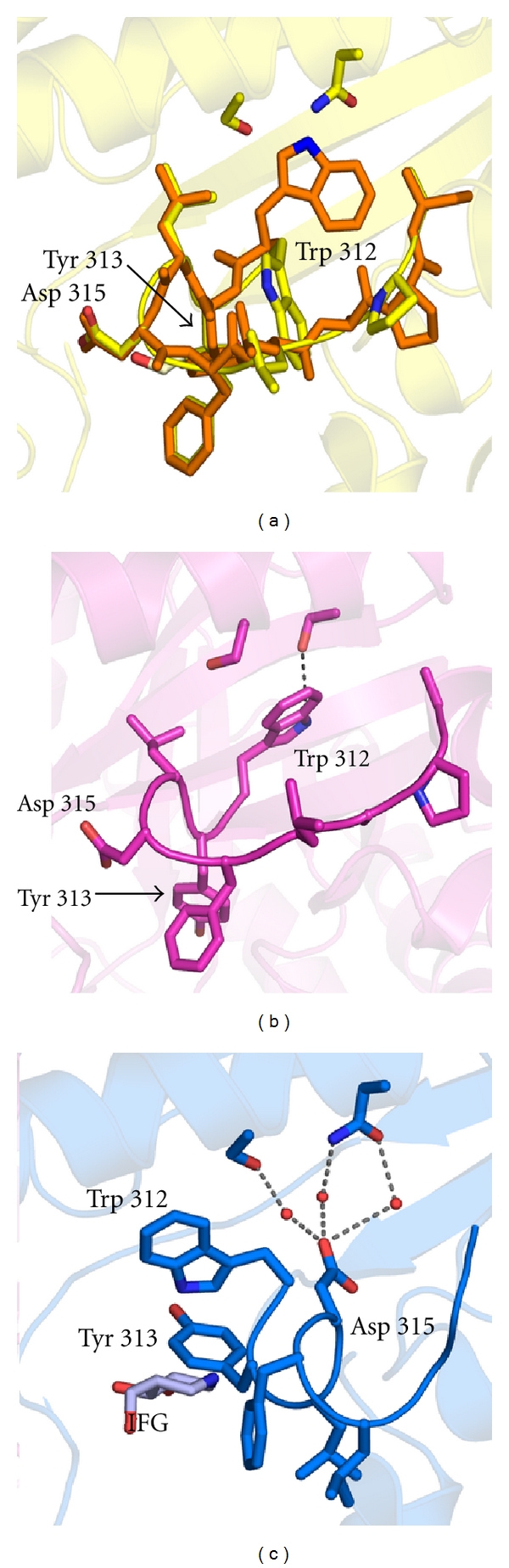
Loop 1 extended and helical conformations: surface view. (a) Extended conformation, overlay of sulfate-bound and glycerol-bound coordinates in this region. (b) Extended conformation found for N370S-mutant GCase (PDB code 3KE0). (c) Helical conformation depicted using coordinates of IFG-bound GCase. Dashed lines are as in Figure 3.

**Figure 6:**
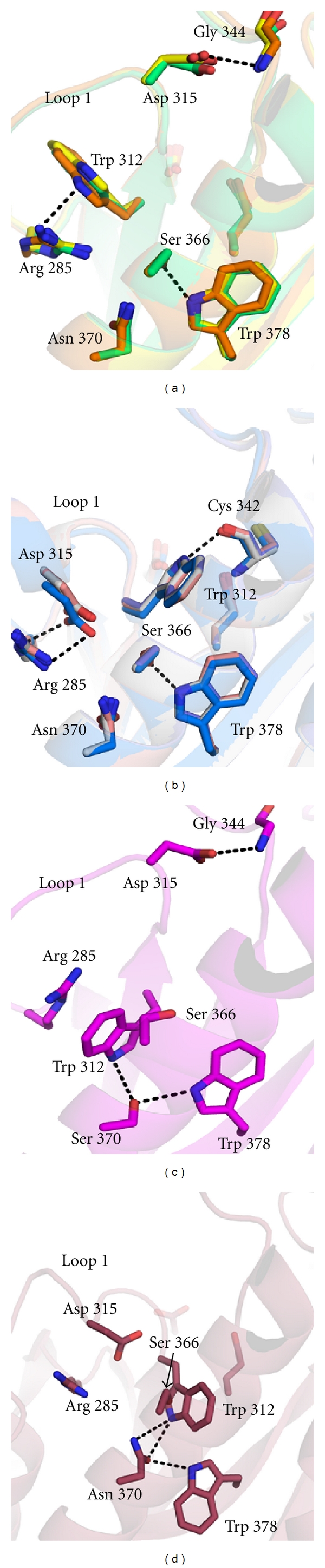
Loop 1 extended and helical conformations: interior view. (a) Extended conformation, overlay of sulfate-bound, glycerol-bound, and CBE-bound coordinates in this region. (b) Helical conformation using coordinates of IFG-bound GCase, NB-DNJ, and NB-DNJ. (c) N370S-mutant GCase. (d) apo-GCase. Dashed lines and color schemes are the same as in Figure 3.

The IFG-bound GCase structure was the first to reveal a substantial rearrangement of Loop 1 from an extended to an *α*-helical turn [35] (Figures 5 and 6). This result was surprising because loop 1 consists of residues that are primarily hydrophobic and thus already match the charge of the expected ceramide moiety in this region. The interplay between the amphipathic residues, Trp 312 and Tyr 313, and the one acidic residue, Asp 315, appears to be key adopting the configurations observed (Figure 5). Asp 315 undergoes the furthest translation in the shift from helical to extended loop 1. In the extended conformation (Figures 5(a) and 5(b)), loop 1 reaches toward loop 2, forming hydrogen-bonding interactions with the main chain of Gly 344 (Figure 6(a)), whereas in the helical conformation, Asp 315 is tucked within the core region of GCase, forming water-mediated hydrogen bonding interactions with the clinically important residue Asp 370 (Figure 5(c)) and a salt bridge with the guanidinium group of Arg 285 (Figure 6(b)). Tyr 313, mentioned earlier with regard to ligand binding in the active site, is also located on this loop, and another residue to participate in hydrogen bonding. Tyr 313 swaps hydrogen bonding partners from Glu 235 to Glu 340 (compare Figure 3(c) to Figure 3(f)). For a ligand to bind in the GCase active site it is tempting to envision an order of events in which a change occurs in the hydrogen bonding pattern of Tyr 313, which in turn disrupts the hydrogen bonding pattern for Asp 315 and enables the loop to take on the new helical configuration. In terms of Trp 312 (Figure 6), in the extended conformation, it is tucked under loop 1 and forms hydrogen bonding interactions with Arg 285 (Figure 6(a)); this is the same relative placement of Asp 315 in the helical turn (Figure 6(b)). In the helical conformation, Trp 312 swings out instead to form a hydrogen bonding interaction with the main chain of Cys 342 (Figure 6(b)). Although the positions of these residues are generally consistent given a loop 1 configuration, two exceptions include the N370S-GCase structure (extended loop 1) [32], in which both Trp 312 and Trp 378 are within hydrogen bonding distance of Ser 370 (Figure 6(c)), and apo GCase (helical loop 1) [38], in which the same tryptophan residues are in contact with Asn 370 (Figure 6(d)).

The helical conformation has been observed in the structures of the DNJ analogs [36], as well as in select apo structures of Cerezyme [38] and Velaglucerase-alfa [30] under different pH conditions, indicating that loop 1 is mobile both at pH 4.5 reminiscent of the lysosome and at higher pH values of the ER. Specifically, in apo structures, half of the molecules in the asymmetric unit are in the extended and other in the helical conformation. It is not currently known whether GCase, which appears to be a functional dimer [70, 71], can only take on one helical conformation at a time, or if this observation is trapped by crystal packing [38]. Comparison of the thermal B-factors reveals that the IFG-bound loop 1 conformation, with its additional secondary structure, is better locked in position than when present as an extended loop [35, 38].

**Figure 7:**
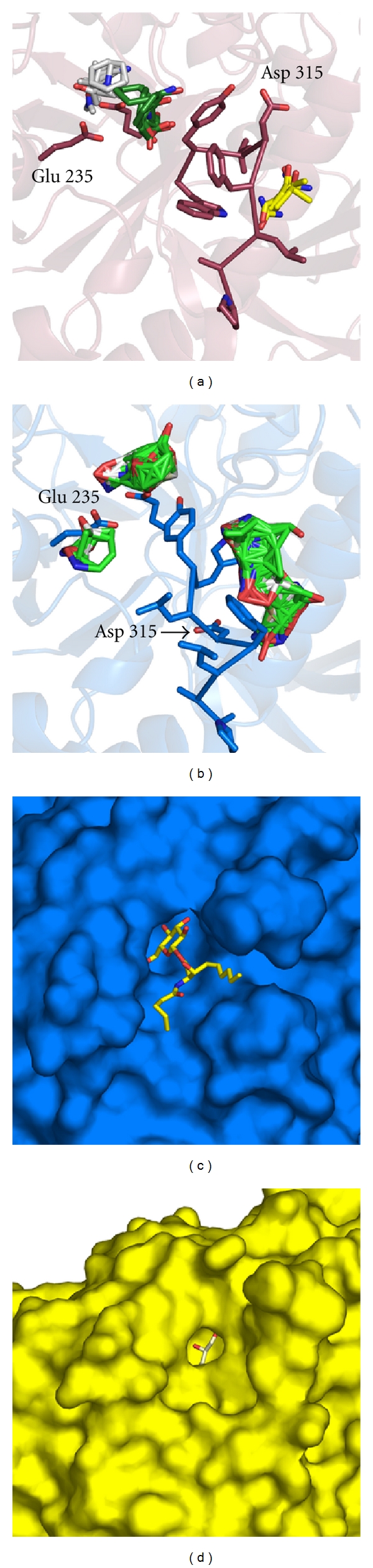
Computational docking and surface representations of GCase. (a) *In silico* fragment drug screening using extended loop 1 (yellow cluster clashes with receptor coordinates). (b) *In silico* fragment drug screening using helical loop 1 (no clashes observed). (c) Surface representation of IFG-bound GCase with ball-and-stick representation of truncated GlcCer computationally docked into the IFG-bound GCase coordinates. (d) Surface representation of glycerol-bound GCase. Glycerol is presented in ball-and-stick to illustrate the limited extent to which the active site is accessible.

The proposal that the configuration of GCase loop 1 observed crystallographically when IFG is bound is the active conformation of GCase is supported by computational docking studies. First, docking of drug fragments onto the original GCase structure with an extended loop 1 predicts a binding site that apparently clashes with this loop 1 arrangement (Figure 7(a)), whereas docking with the helical loop 1 results in a similar cluster but without clashes (Figure 7(b)) [72]. This result is especially notable in that the algorithm used for docking provides for only minimal perturbation in the receptor coordinates [72]. Second, simulations of GlcCer docking using a truncated ceramide to limit degrees of freedom place the hydrophobic tails in the two subsites emerging from the catalytic center. A reasonable pose, in which the glucose head group is well positioned with respect to the catalytic residues, is only observed when using a receptor with helical loop 1 [35] (Figure 7(c)). The glucose moiety is not properly positioned when the extended loop 1 is present in the receptor. A surface representation of this receptor reveals that the active site covers the much smaller glycerol molecule and thus is inaccessible to larger ligands (Figure 7(d)). 

### 2.6. Effects of Disease-Causing Mutants

Prior to the observation of the loop 1 helical conformation, it was difficult to reconcile how Asn 370, a residue 13 Å from the catalytic glutamate residues, could reduce enzymatic activity by nearly 80% [16]. With the N370S-mutant GCase structure in hand, we now know that only the extended loop 1 is observed at both acidic and neutral pH [32]. Overall, the structure is more rigid and exhibits minor stability changes compared to wild-type GCase with no pH-dependent changes observed in structure or circular dichroism spectrum [32]. As expected for the extended loop, Tyr 313 is hydrogen bonded to Glu 235, but some changes are observed in the interior region, as described above, with Trp 312 and Trp 378 (Figure 6(c)). The implications of this hydrogen bonding shift are not clear, but given that this amino acid change is accompanied by decreased enzyme activity and a disease state, the apparent preference of an extended loop 1 in N370S-GCase is likely intimately related to efficiency of GCase catalysis.

Among the mutants selected for highlight in Figure 2, N370S is the best understood to date. Due to the discontinuous nature of the domains with respect to amino acid sequence, disease-causing residues close in sequence can be found in different GCase domains, with different consequences. Mysteries remain for mutations such as G202R, D409H, and L444P, which lead to different manifestations of neuropathogenic GD. In the case of Gly 202, located on a surface loop of Domain 2, there appears to be no immediate shape or charge constraints. Located on a short helical segment between two strands of Domain 1, Asp 409 participates in hydrogen bonding interactions with the backbone nitrogen and side chains of Ser 97. At first glance, it would appear that histidine could participate in similar interactions but upon closer inspection, the presence of adjacent proline residues, Pro 98-99, suggests that this interacting loop is rather rigid. Leu 444 is located on a loop within Domain 3 and is involved in hydrophobic interactions with a cluster of leucines in its vicinity. Mutation to a proline would be expected to rigidify the protein backbone and perhaps propagate to a significant new location for another residue on the loop, such as Ser 439, which is involved in both main chain and side chain interactions with a nearby strand. Overall, it is not possible to predict severity of disease based on location in GCase, nor is the effect readily rationalized based on the chemical environment of the residue. Solution biophysical studies and additional structures of disease-relevant mutant GCases would assist in understanding structural and stability defects that may contribute to disease.

### 2.7. Glycosylation Sites

The endogenous human GCase enzyme is glycosylated at 4 of 5 available asparagine residues, and glycosylation is important for the formation of the active enzyme [73]. Cerezyme, Velaglucerase-alfa, and prGCase have different glycans due to their different manufacturing processes and their engineering for targeting to and uptake by macrophages using the mannose receptor pathway. The carrot-cell-expressed prGCD exhibits unique, plant-derived glycan cores, including *α*-(1,2)-xylose and *α*-(1,3)-fucose. The enzyme is targeted to the vacuole, leading to GCase with terminal mannose [29]. To enhance its internalization by macrophages, the CHO-cell-derived Cerezyme is sequentially delgycosylated to leave the core glycan consisting of 2 N-acetyl-glucosamine and 3 mannose sugars [3]. In the crystal structure of Cerezyme not subjected to treatment by N-glycosidase F [34], five sugars are observed attached to Asn 19, three on Asn 59, and two on Asn 146. No sugars are observed bound to Asn 270 likely due to disorder in the crystal [34], nor Asn 462, a buried residue confirmed earlier to lack glycosylation [73]. High resolution mass spectrometry data reveals that Cerezyme contains ~0.6 mole M6P per enzyme and core structures that terminate in N-acetyl-glucosamine, as well as some microheterogenetity at Asn 59, Asn 146, and Asn 270 that includes fucosylation and phosphorylation of high mannose carbohydrates [30]. Similar analysis of Velaglucerase-alfa reveals 0.8 mole of M6P and predominantly nine mannose units. At Asn 59, Asn 146, and Asn 270, mono-siaylation and complex type structures with core fucosylation, as well as phosphorylation, were also observed at lower levels [30]. Compared to Cerezyme, Velaglucerase-alfa is internalized to macrophages 2.5-fold faster, likely a result of the different glycosylation patterns [30].

### 2.8. Anion Binding Sites

**Figure 8:**
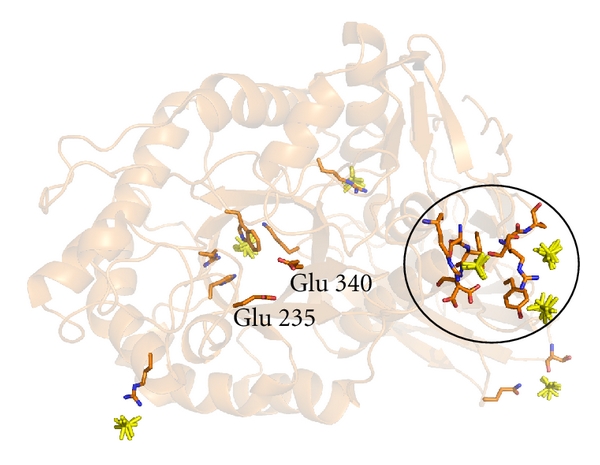
Anion binding sites on GCase. Sulfate or phosphate anions are presented in yellow ball-and-stick; interacting residues are presented in orange; active site residues Glu 235 and Glu 340 are labeled as well. Circled: cluster of several anions that may represent an anionic lipid binding site.

GCase is associated with the lysosomal membrane in vivo [74], and negatively charged phospholipids are required for optimal activity in vitro [28, 31, 74], suggesting that specific binding sites for anions may be present on GCase. Several such binding sites can be inferred from bound phosphate and sulfate anions modeled in the solved structures, which arise from the salts used in the crystallization solution. In particular, among the structures of Cerezyme, Velaglucerase-alfa, and prGCase, there are seven apparent anion binding sites (Figure 8). A particular cluster of note contains three binding sites, corroborated among the various structures and is found on Domain 3 on the same face as the GCase active site in Domain 2. The anions are held in place by Ser 12, Ser 23, Arg 44, Arg 353, Ser 356, Tyr 487, and the backbone nitrogens of Ser 45, Trp 357, and Asp 358 (Figure 8 circled). This site may be important for phospholipid binding and membrane association [34]. The other anion binding sites, scattered on the GCase surface, appear to have just one anion bound, suggesting that these may be nonspecific binding sites.

## 3. Structure of Saposin C

### 3.1. Protein Sources

The structure of SapC has been determined by both NMR and X-ray crystallographic techniques using recombinant SapC purified from *E. coli* [45, 75] or *P. pastoris* [50, 76]. Since the writing of the most recent review article dedicated to saposin structure [77], several new structures of SapC have arisen using both techniques under different chemical environments.  Table 2 summarizes the available SapC structures.

### 3.2. Structure Determination

The first solution NMR structure of SapC was solved at pH 4 and pH 7 using a suite of heteronuclear NMR experiments to determine distance restraints and dipolar couplings [45]. The subsequent solution structure was determined by similar methods in the presence of 25 mM sodium dodecyl sulfate (SDS), perdeuterated as necessary. The coordinates of the first NMR structure were used as a molecular replacement model to solve the first 2 Å resolution crystal structure. These crystals were grown from a solution at pH 6 or 7 and belong to the hexagonal space group P6_3_ (approximate unit cell dimensions *a*=*b*= 53 Å, *c* = 52.5 Å, **β** = 120°, and one molecule in the asymmetric unit) [78]. Additional crystal forms of SapC have been solved at pH 5 [50]: tetragonal (P4_1_2_1_2, unit cell dimensions *a*=*b*= 49 Å, *c* = 155.6 Å, all angles 90°, 2 molecules in asymmetric unit) and orthorhombic (C222_1_, unit cell dimensions 57, 89, 93.5 Å, all angles 90°, 2 molecules in asymmetric unit), using coordinates of the first reported crystal structure.

### 3.3. Overall Structure

**Figure 9:**
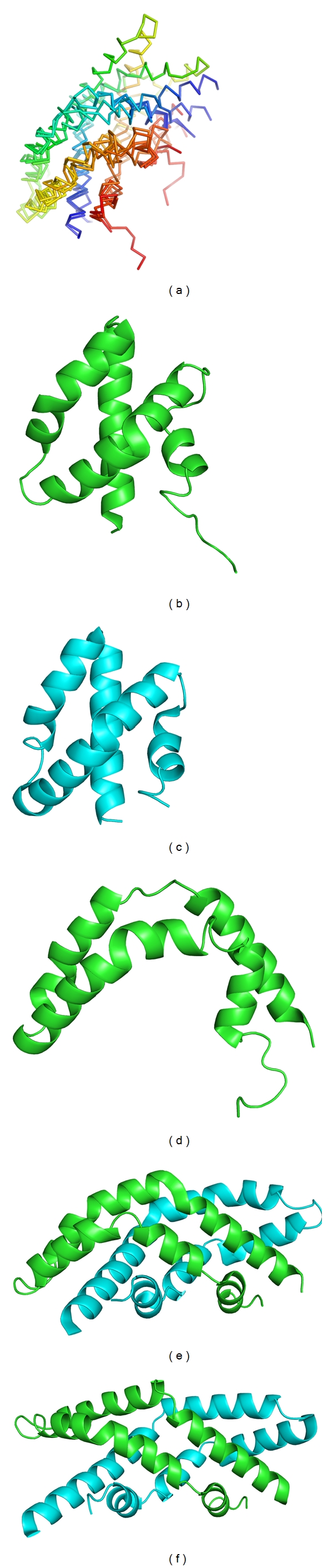
Structures of SapC. (a) Overlay of structures presented in (b)–(f) colored in a rainbow from N-terminus (blue) to C-terminus (red). (b) Closed NMR structure (PDB code 1M12). (c) Closed crystal structure (PDB code 2GTG). (c) Open NMR structure (PDB code 1SN6). (d) Open crystallographic dimer (orthorhombic, PDB code 2Z9A). (e) Open crystallographic dimer (tetragonal, PDB code 2QYP).

Solution NMR [45, 75] and X-ray crystal structures [50, 78] reveal a flexible SapC (Figure 9(a)) composed of 4 or 5 amphipathic helices, two pairs of which are disulfide bonded. SapC adopts two main configurations: (a) “closed” helical bundle (Figures 9(b) and 9(c)) and (b) “open,” boomerang shape with a range of obtuse hinge angles (Figures 9(d)–9(f)) [50] that reveal a hydrophobic surface. Lipid binding to SapC is not fully understood. Lipids are proposed to bind only after neutralization of the negative electrostatic surface by a pH-controlled reversible process [45, 76] but which, if any, of the available structures is biologically functional is unclear. For the SapC monomer, no structural change was detected by NMR upon binding to phospholipid vesicles [45], but an open structure was observed with the addition of SDS [75]. To complicate matters, SapC has been shown to be a dimer [76] and trimer in solution at low pH [78], and two of the available crystal structures solved near neutral pH are the domain-swapped dimers (Figure 9) [50]. The bundled monomer and dimeric species shield a hydrophobic surface; one method to elicit a conformational change upon lipid binding would be via its interactions with positively charged lysine residues that would propagate to expose this hydrophobic surface. Alternatively, the extended dimer could be functional, with each end participating in membrane interactions [50, 79].

## 4. Complex of GCase and Saposin C

**Figure 10:**
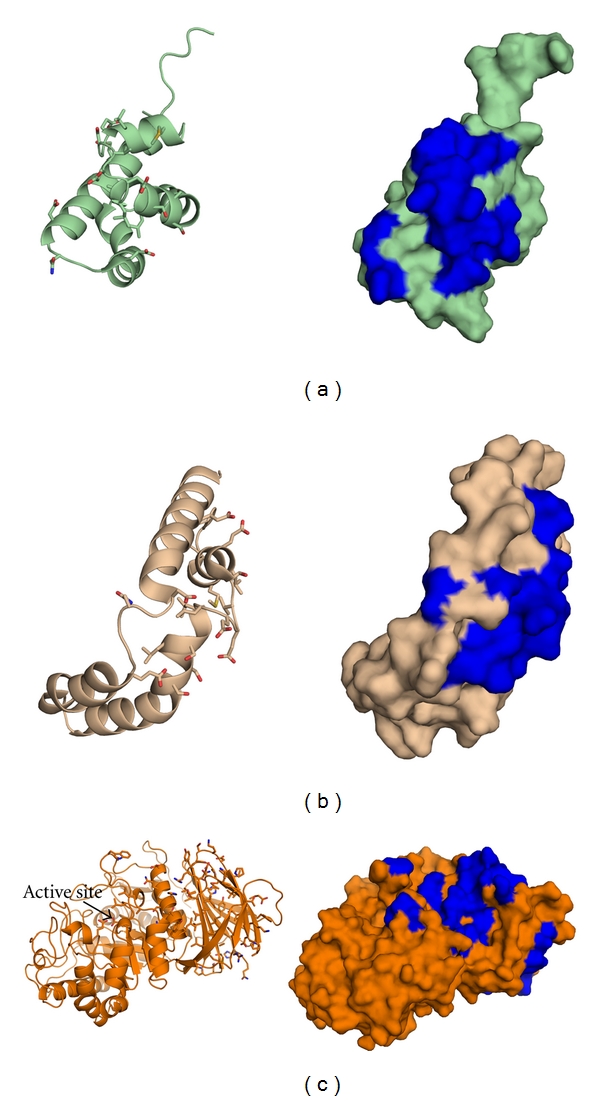
Interaction surfaces for GCase-SapC complex identified by computational docking. (a) Closed NMR structure, (b) open NMR structure, and (c) sulfate-bound GCase each with proposed interacting residues in ball-and-stick (left) and highlighted blue in surface representation on right.

In spite of the evidence that stresses the importance of SapC for GCase enzyme activity and their genetic mutations that lead to GD, the specific site of their presumed interaction has not been explicitly established. Work towards understanding the interaction includes experiments localizing the SapC binding site in the proximity of N370 [80], investigating interactions with site-directed mutants of SapC [49, 81] and through investigations using model peptides derived from the SapC sequence [82]. A computation docking model [83] utilizing the closed or open SapC coordinates from NMR (PDB code 1M12 or 1SN6, resp.) and those corresponding to the first GCase structure (extended loop 1, PDB code 1OGS) reveals a localized surface that includes interactions from both domains 1 and 2 of GCase and a cluster on SapC (Figure 10). This model correlates reasonably well with experimental findings that a peptide composed of residues 41–82 binds best to GCase [82] and that residues important for GCase activation are localized to residues 47–62. Similar computational docking calculations with GCase in its active conformation might provide additional strength to the identification of this binding interface.

## 5. Future Directions

The structures of GCase and SapC have been very valuable to confirm experimental observations. Assisted by computational modeling of interactions, new structure-based hypotheses for additional experiments, as well as inspire rational drug design and discovery have emerged. Still, many questions remain to be addressed by solution biophysical and structural studies, which include, but are not limited to the following.

- Lysosomal Trafficking: is LIMP-II the universal lysosomal chaperone for GCase? If so, it is likely that a stable interaction forms between these two proteins, and characterization of the complex could provide new insight into ways to favor trafficking of mutant GCases over their degradation by stabilizing the interaction with LIMP-II.
- Structural aspects of GCase: is the helical loop 1 as critical for catalysis as proposed? Structures with nonhydrolysable substrate analogs or additional inhibitors could continue to provide insight into GLCase catalysis, the plasticity of the GCase active site, and ways in which remotely located mutations could impair enzyme activity.
- Effects of mutations on GCase: why are certain mutations pathogenic if they yield enzymes in vitro? Both structural and modeling studies of specific mutants could provide additional insight into these defects.
- Structural aspects of SapC: to what extent are the available structures functionally relevant? How does lipid bind and what is the mechanism of solubilization by SapC? The characterization additional constructs, such as those containing disease-causing mutations, may provide new insight into the flexibility of SapC upon lipid binding.
- Complex between GCase and SapC: what is the affinity of these proteins for each other? What components, such as lipids, substrate, or membrane, might also be critical to detect as stable interaction, if formed? With all of the components in place it may be possible to isolate a complex for structure determination. If the interaction is transient, what is the rationale?

## Figures and Tables

**Table 1:** Crystal structures reported for GCase.

PDB code	Enzyme source	Deglycosylated?	Active site	Crystallization condition	pH	Ref.
1OGS	CHO/Cerezyme	PD^a^	Sulfate	Ammonium sulfate, Guanidinium HCl, KCl, acetate buffer, cryoprotected with glycerol	4.6	[24]
1Y7V	CHO/Cerezyme	PD	CBE	Same as 1OGS, soaking overnight with 1 mM CBE, cryoprotected with glycerol	4.6	[33]
2F61	CHO/Cerezyme	PD		Ammonium sulfate, citrate buffer, magnesium chloride	6	[16]
2J25	CHO/Cerezyme	No		Ammonium sulfate, bis-tris buffer	5.5	[34]
2NSX	CHO/Cerezyme	PD	IFG	Same as 1OGS, soaking for 10′ with 0.2 mM IFG	4.5	[35]
2NT0	CHO/Cerezyme	PD	Glycerol	Same as 1OGS	4.5	[35]
2NT1	CHO/Cerezyme	PD		Na, K Dihydrogen phosphate, Hepes buffer, lithium sulfate cryoprotectant	7.5	[35]
2V3D	Plant/Taliglucerase-alfa	No	NB-DNJ	Ammonium sulfate, Tris buffer, PEG 3350; cocrystallization with ligand	6.5	[36]
2V3E	Plant/Taliglucerase-alfa	No	NN-DNJ	Ammonium acetate, Hepes buffer, PEG 3350; cocrystallization with ligand	7.5	[36]
2V3F	Plant/Taliglucerase-alfa	No	N/A	Ammonium sulfate, bis-Tris buffer, hexamine cobalt(III) chloride, PEG 3350	6.5	[29]
2VT0	Plant^1^	N/A^b^	N/A	Ammonium sulfate, Tris buffer, PEG 3350	6.5	N/A
2WCG	Plant/Taliglucerase-alfa	No	N-octyl(cyclic guanidine)-nojirimycin	Same as 2V3D, cocrystallization with ligand	6.5	[37]
2WKL	Human cell line/Velagucerase-alfa	No		Ammonium sulfate, Hepes buffer, PEG 8000, ethylene glycol cryoprotectant	7	[30]
3GXD	CHO/Cerezyme	PD		Na, K Dihydrogen phosphate, acetate buffer, lithium sulfate cryoprotectant	4.5	[38]
3GXF	CHO/Cerezyme	PD	IFG	Na, K Dihydrogen phosphate, Hepes buffer, glycerol cryoprotectant, soaking for 10′ with 0.5 mM IFG	7.5	[38]
3GXI	CHO/Cerezyme	PD		Na, K Dihydrogen phosphate, citrate buffer, lithium sulfate cryoprotectant	5.5	[38]
3GXM	CHO/Cerezyme	PD		Same as 1OGS	4.5	[38]
3KE0	Baculovirus (N370S-GCase)	PD		Same as 1OGS	5.4	[32]
3KEH	Baculovirus (N370S-GCase)	PD		Na, K Dihydrogen phosphate, Hepes buffer, glycerol cryoprotectant	7.4	[32]

^a^PD: partially glycosylated

^b^N/A: Not applicable or not available.

**Table 2:** Structures of saposin C.

PDB code	Enzyme source	Detergent?	Technique	Experimental summary	Citation
1M12	*E. coli*	No	NMR	^15^N, ^1^H, and ^13^C heteronuclear NMR experiments	[45]
1SN6	*E. coli*	Yes, SDS	NMR	^15^N, ^1^H, and ^13^C heteronuclear NMR experiments	[75]
2GTG	*E. coli*	No	Crystallography	Calcium chloride, Hepes buffer pH 7 or cacodylate buffer pH 6, glycerol cryoprotectant	[78]
2QYP	*P. pastoris*	No	Crystallography	Magnesium sulfate or ammonium sulfate, acetate buffer pH 4, pentaerythritol ethoxylate 15/4	[50]
2Z9A	*P. pastoris*	No	Crystallography	Same as 2QYP	[50]
